# Objective assessment of bradykinesia in Parkinson’s disease using evolutionary algorithms: clinical validation

**DOI:** 10.1186/s40035-018-0124-x

**Published:** 2018-08-16

**Authors:** Chao Gao, Stephen Smith, Michael Lones, Stuart Jamieson, Jane Alty, Jeremy Cosgrove, Pingchen Zhang, Jin Liu, Yimeng Chen, Juanjuan Du, Shishuang Cui, Haiyan Zhou, Shengdi Chen

**Affiliations:** 1 Department of Neurology, Ruijin Hospital, Shanghai Jiao Tong University School of Medicine, Shanghai, China; 2 Department of Electronic Engineering, University of York, York, UK; 3 Department of Computer Science, Heriot-Watt University, Edinburgh, UK; 4 Department of Neurology, Leeds General Infirmary, Leeds Teaching Hospitals NHS Trust, Leeds, UK

**Keywords:** Parkinson’s disease, Bradykinesia, Evolutionary algorithms, Objective assessment, Clinical validation

## Abstract

**Background:**

There is an urgent need for developing objective, effective and convenient measurements to help clinicians accurately identify bradykinesia. The purpose of this study is to evaluate the accuracy of an objective approach assessing bradykinesia in finger tapping (FT) that uses evolutionary algorithms (EAs) and explore whether it can be used to identify early stage Parkinson’s disease (PD).

**Methods:**

One hundred and seven PD, 41 essential tremor (ET) patients and 49 normal controls (NC) were recruited. Participants performed a standard FT task with two electromagnetic tracking sensors attached to the thumb and index finger. Readings from the sensors were transmitted to a tablet computer and subsequently analyzed by using EAs. The output from the device (referred to as "PD-Monitor") scaled from − 1 to + 1 (where higher scores indicate greater severity of bradykinesia). Meanwhile, the bradykinesia was rated clinically using the Movement Disorder Society-Sponsored Revision of the Unified Parkinson’s Disease Rating Scale (MDS-UPDRS) FT item.

**Results:**

With an increasing MDS-UPDRS FT score, the PD-Monitor score from the same hand side increased correspondingly. PD-Monitor score correlated well with MDS-UPDRS FT score (right side: *r* = 0.819, *P* = 0.000; left side: *r* = 0.783, *P* = 0.000). Moreover, PD-Monitor scores in 97 PD patients with MDS-UPDRS FT bradykinesia and each PD subgroup (FT bradykinesia scored from 1 to 3) were all higher than that in NC. Receiver operating characteristic (ROC) curves revealed that PD-Monitor FT scores could detect different severity of bradykinesia with high accuracy (≥89.7%) in the right dominant hand. Furthermore, PD-Monitor scores could discriminate early stage PD from NC, with area under the ROC curve greater than or equal to 0.899. Additionally, ET without bradykinesia could be differentiated from PD by PD-Monitor scores. A positive correlation of PD-Monitor scores with modified Hoehn and Yahr stage was found in the left hand sides.

**Conclusions:**

Our study demonstrated that a simple to use device employing classifiers derived from EAs could not only be used to accurately measure different severity of bradykinesia in PD, but also had the potential to differentiate early stage PD from normality.

## Background

Parkinson’s disease (PD) is the second most common neurodegenerative disorder and is characterized by bradykinesia, resting tremor and rigidity. It has affected approximately 1.7% of the population over 65 years old in China and has a profound impact on the patients’ daily lives [1]. Although currently there is no cure for PD, the correct diagnosis is important not only for the treatment and prognosis, but also for clinical trials and epidemiological studies. However, practically misdiagnosis of PD is much common. A recent review evaluated the accuracy of clinical diagnosis of PD reported in the last 25 years and found that it was only 80.6% [2]. Accuracy has not significantly improved in the last 25 years, particularly in the early stages of the disease. Essential tremor (ET) is one of the conditions most easily confused with PD, especially in their early stages where clinical signs are subtle [3]. Although various types of tremors are the major clinical overlaps between these two disease entities, a number of ET patients also present with bradykinesia and gait disturbance mimicking the symptoms of PD [4, 5].

Bradykinesia is the prerequisite for PD diagnosis [6], which makes accurate identification of bradykinesia pivotal. It can be evaluated by a neurologist’ subjective judgment of several tasks, such as finger tapping (FT), hand movements, pronation-supination movements, toe tapping and foot tapping [7]. Unfortunately, bradykinesia-related items have the lowest reliability among all Unified Parkinson’s Disease Rating Scale (UPDRS) III items (motor evaluation), particularly when the severity is slight or mild [8]. Therefore, there is an urgent need to develop objective, effective and convenient measurements to help clinicians accurately identify bradykinesia, which can potentially lead to earlier diagnosis of PD.

Recently, a United Kingdom (UK)-based research group has developed a system called *PD-Monitor* that employs the evolutionary algorithm (EA, a form of artificial intelligence) to induce classifiers capable of recognizing bradykinesia in PD patients when performing FT tasks [9]. The aim of this study was to a) validate the accuracy of the EA to assess bradykinesia of FT in a Chinese cohort, b) examine the ability of the EA to distinguish early stage PD patients, and c) explore whether the EA scores related with disease severity.

## Subjects and methods

### Subjects

A total of 107 PD patients, 41 ET patients and 49 age and gender matched normal controls (NC) participated in this study. Normal controls were recruited from patients’ spouses and companions. In order to rule out data bias due to dominant hand difference, all selected participants were right-hand dominant. The diagnosis of PD or ET was confirmed by two or more experienced movement disorder specialists, according to the MDS clinical diagnostic criteria for PD [6] and diagnostic criteria for ET [10]. Exclusion criteria included: cognitive impairment (Mini Mental State Examination (MMSE) < 24 [11]), clinically defined fracture or arthritis of an upper limb, and other central nervous system diseases that might affect hand flexibility. All the PD patients were treated with anti-parkinsonism medication and at “on” medication status when assessed except for 17 de novo patients. There were 10 PD patients whose FT bradykinesia was not obvious and scored zero when they were at “on” medication status. They were only included in the evaluation of the correlation between PD-Monitor FT objective score and MDS-UPDRS FT subjective score. The study was approved by the Ethics Committee of Ruijin Hospital, Shanghai Jiao Tong University School of Medicine. Written informed consents were obtained from all the participants.

## Methods

Demographic data and medical history were collected from all participants. Cognitive function was evaluated using the MMSE [12] and disease severity was evaluated using the modified Hoehn and Yahr (H-Y) scale [13]. The total daily dose of dopaminergic medications in each patient was determined by means of L-dopa equivalent daily dose (LEDD, mg/day) based on theoretical equivalence as previously reported [14].

### Assessment of finger-tapping

**Fig. 1:**
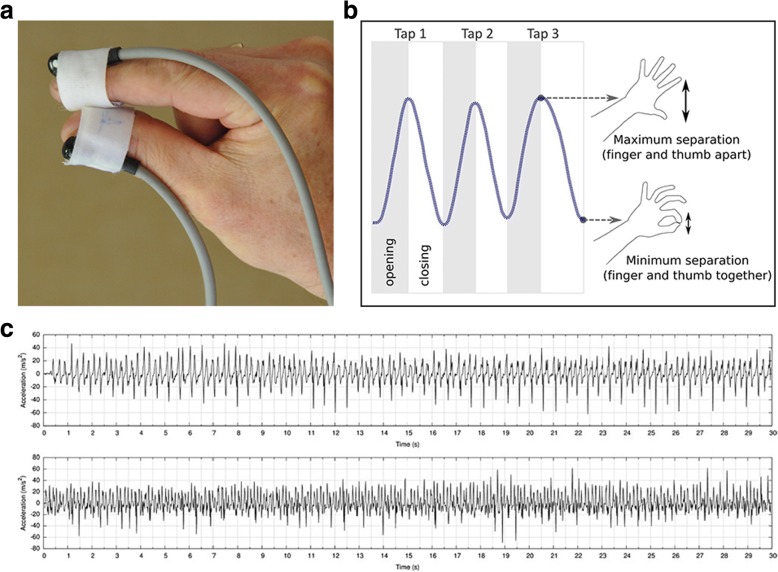
Schematic representations of sensors and finger tapping task. **a** Sensors attached to nail bed of index finger and thumb. **b** Opening and closing phases of the finger tapping task. **c** Example data from two patients showing acceleration of fingers during the finger tapping task

FT data were collected using two small, simple and non-invasive sensors which were attached to the subject’s thumb and index finger while performing FT tasks (Fig. 1a). The sensors have a sampling rate of 60 Hz, and measure both position and orientation relative to a point source in real time. Readings from the sensors are transmitted to a tablet computer and then analyzed by the specialist software employing EA. These algorithms have been trained to recognize PD patients’ movements characteristic of bradykinesia [9]. The calculated value based on the FT movement pattern is then presented on the tablet, using an objective score scaled from − 1 to + 1 (where higher scores indicate greater severity of bradykinesia). The EA device is referred as “PD-Monitor” hereafter.

Finger Tapping: FT is a standard clinical test for evaluating bradykinesia. The subjects were asked to bend their elbow and raise their hand, making sure that the arm was unsupported. They were then instructed to tap their thumb and index finger repeatedly for 30 s as rapidly as possible, separating the finger and thumb as far as possible [9]. They were allowed to rest for 1 min before repeating the task with the same hand. The larger PD-Monitor score of the two exercises was used for further statistical analysis. FT data were recorded from each hand. Figure 1b illustrated the finger tapping process, and Fig. 1c showed the example data acquired from two patients, presented graphically as the acceleration of the fingers over the duration of the FT task.

Movement Disorder Society-sponsored revision of the UPDRS (MDS-UPDRS) Ratings: During FT tasks, the bradykinesia of both hands were independently rated by two neurologists who were blinded to the PD-Monitor score using the “finger-tapping” item of the MDS-UPDRS III (item 3.4). FT was scored from 0 to 4. Zero corresponds to normal action and 4 indicates that the task could hardly be performed [7]. Rating discrepancies were discussed between the raters, and then settled by an agreed score. We used the MDS-UPDRS FT score (item 3.4) as a gold standard for abnormality or slowness of FT.

### Evolutionary algorithms

EAs are a form of artificial intelligence that provide a generic method for optimizing classifier models to fit data. The algorithm refines a population of classifiers through a repeated process of variation and selection inspired by the theory of Darwinian evolution. Selection is based on maximizing fitness criteria, the area under the receiver operating characteristic (ROC) curve (AUC), when discriminating the FT movements of PD patients from NC in a training data set. These movement recordings were gathered during a clinical study held at a neurology center in the UK [9, 15]. EAs are stochastic, meaning that different solutions are found each time the algorithm is executed. To address this, the best performing classifier is selected from 50 repeated runs of the algorithm. A classifier takes the form of a symbolic equation that is applied to a movement data sequence using a standard sliding window approach. Inputs to the classifier are the individual accelerations within each window. The output of the classifier is the mean of the symbolic equation’s output across all windows in a sequence. Full details of the implementation of the algorithm can be found in M. A. Lones et al.’s research [9].

### Statistical analysis

The statistical analyses were conducted using SPSS software v.22.0 [16]. Data were expressed as numbers or as mean ± SE. Nonparametric Kruskal-Wallis one-way analysis of variance with post hoc tests as needed were used in all data analysis procedures. Mann-Whitney U test and the Chi-squared test were used for continuous variables or categorical variables. ROC curves were constructed using PD-Monitor scores from PD and NC. Spearman rank-order correlation was used to evaluate the association. Partial correlation was used to further examine the correlation of PD-Monitor score and modified H-Y stage after controlling age, gender, disease duration and LEDD. The limit of significance was set at *P* < 0.05 (two-tailed).

## Results

### PD-monitor FT objective score correlated well with the MDS-UPDRS FT subjective score

The ability of the EA to detect bradykinesia and its severity was assessed in 107 PD patients and 49 NC. Demographic and clinical data of all participants were demonstrated in Table 1. All the subjects were classified into four subgroups according to their MDS-UPDRS FT grade from 0 to 3 (no patient scored 4 in our study). A total of 312 assessments of both hands were recorded, where 65 of the PD FT assessments were scored 0, 49 scored 1, 63 scored 2, and 37 scored 3, and all 98 of the NC FT assessments were scored 0.

**Table 1:** Demographic and clinical data of the study participants

	PD	NC	ET	*P* value
Overall subjects				
Number	*N* = 107	*N* = 49	*N* = 41	
Gender[male/female]	48/59	18/31	18/23	0.625
Age[years]	62.1 ± 0.8	61.9 ± 1.2	60.0 ± 1.9	0.515
Disease duration[years]	5.0 ± 0.4	NA	9.8 ± 1.3	0.003^**^
LEDD (mg)	311.2	NA	NA	
Subjects with right affected side				
Number	*N* = 74	*N* = 49		
Gender[male/female]	33/41	18/31		0.386
Age[years]	62.4 ± 1.0	61.9 ± 1.2		0.662
Subjects with left affected side				
Number	*N* = 75	*N* = 49		
Gender[male/female]	32/43	18/31		0.510
Age[years]	62.8 ± 1.0	61.9 ± 1.2		0.490
Subgroup of PD (FT = 1) vs.NC				
Subjects with right affected side				
Number	*N* = 19	*N* = 49		
Gender[male/female]	7/12	18/31		0.993
Age[years]	61.9 ± 2.1	61.9 ± 1.2		0.995
Subjects with left affected side				
Number	*N* = 30	*N* = 49		
Gender[male/female]	13/17	18/31		0.560
Age[years]	62.3 ± 1.7	61.9 ± 1.2		0.705
Subgroup of PD (FT = 2) vs.NC				
Subjects with right affected side				
Number	*N* = 38	*N* = 49		
Gender[male/female]	18/20	18/31		0.318
Age[years]	63.6 ± 1.3	61.9 ± 1.2		0.389
Subjects with left affected side				
Number	*N* = 25	*N* = 49		
Gender[male/female]	8/17	18/31		0.687
Age[years]	66.1 ± 1.5	61.9 ± 1.2		0.051
Subgroup of PD (FT = 3) vs.NC				
Subjects with right affected side				
Number	*N* = 17	*N* = 49		
Gender[male/female]	8/9	18/31		0.453
Age[years]	60.5 ± 2.3	61.9 ± 1.2		0.809
Subjects with left affected side				
Number	*N* = 20	*N* = 49		
Gender[male/female]	11/9	18/31		0.163
Age[years]	59.4 ± 1.7	61.9 ± 1.2		0.334
Subgroup of PD (H-Y = 1) vs.NC				
Subjects with right affected side				
Number	*N* = 18	*N* = 49		
Gender[male/female]	8/10	18/31		0.566
Age[years]	60.2 ± 2.0	61.9 ± 1.2		0.457
Subjects with left affected side				
Number	*N* = 18	*N* = 49		
Gender[male/female]	8/10	18/31		0.566
Age[years]	59.3 ± 1.9	61.9 ± 1.2		0.311

Kruskal-Wallis test and Mann-Whitney U test were used to compare the continuous variables. The Chi-square test was used to compare the categorical variables. Data were expressed as numbers or as mean ± SE. ^**^ *P* < 0.01

*PD* Parkinson’s disease, *ET* essential tremor, *NC* normal controls, *H-Y* modified Hoehn and Yahr stage, *LEDD* levodopa equivalent daily dose, *NA* not available

**Fig. 2:**
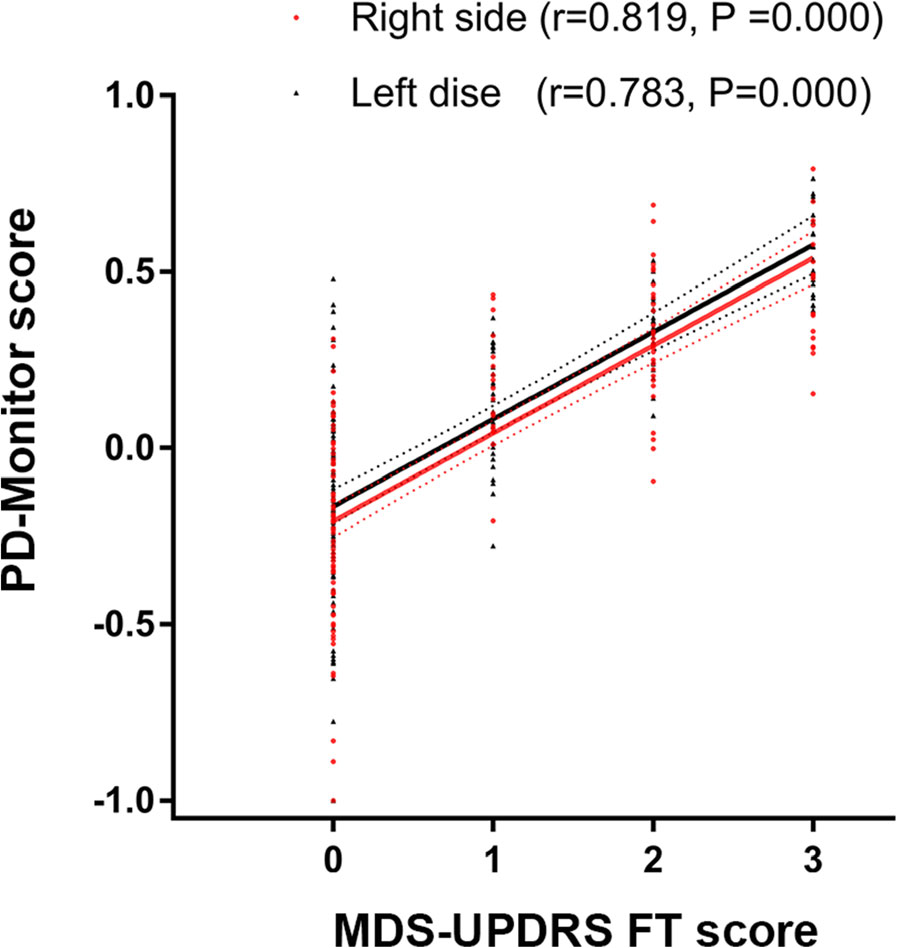
PD-Monitor FT objective score correlated well with the MDS-UPDRS FT subjective score. Spearman rank-order correlation analysis revealed that the PD-Monitor score positively correlated with MDS-UPDRS FT score. Right side: *r* = 0.819, *P* = 0.000. Left side: *r* = 0.783, *P* = 0.000. MDS-UPDRS, Movement Disorder Society-sponsored revision of the Unified Parkinson’s disease rating scale; FT, finger tapping

It demonstrated that with an increasing MDS-UPDRS FT score, the PD-Monitor score from the same hand side increased correspondingly (right side: 0: − 0.23 ± 0.03; 1: 0.17 ± 0.04; 2: 0.32 ± 0.03; 3: 0.46 ± 0.04, *P* = 0.000; left side: 0: − 0.17 ± 0.03; 1: 0.12 ± 0.03; 2: 0.33 ± 0.02; 3: 0.56 ± 0.03, *P* = 0.000). Post hoc tests revealed a significant difference in PD-Monitor score between subjects with FT scored 0 and other subgroups (all with *P* = 0.000) for the right sides. For the left sides, the significant difference was also found between subjects with FT scored 0 and other subgroups (all with *P* ≤ 0.002), and between subjects with FT scored 1 and 2 (*P* = 0.046), or 3 (*P* = 0.000). Spearman rank-order correlation analysis further revealed that the PD-Monitor score correlated well with MDS-UPDRS FT score (right side: *r* = 0.819, *P* = 0.000; left side: *r* = 0.783, *P* = 0.000, see Fig. 2).

### PD-monitor FT objective score detected different severity of bradykinesia

There were 10 out of 107 PD patients whose FT bradykinesia of both hands was not obvious and scored zero when assessed at ‘on’ medication status, and they were excluded for the further analysis. The remaining 97 PD were further classified into different subgroups based on their MDS-UPDRS FT scores and affected hand sides. Seventy-four PD patients exhibited detectable bradykinesia with their right hands, among them 19 were scored 1, 38 scored 2 and 17 scored 3. For the left hands, 75 patients showed detectable bradykinesia, among them 30 were scored 1, 25 scored 2 and 20 scored 3. The overall 97 PD patients and the different subgroups (FT = 1, FT = 2 and FT = 3) were compared with 49 age and gender matched NC with their same hand sides, respectively. Clinical and demographic data of subjects were shown in Table 1.

It demonstrated that PD-Monitor scores in overall PD patients and each PD subgroup were all higher than that in NC (right affected side: 0.31 ± 0.02, 0.17 ± 0.04, 0.32 ± 0.03, 0.46 ± 0.04 vs. -0.24 ± 0.03, all with *P* ≤ 0.001; left affected side: 0.31 ± 0.03, 0.12 ± 0.03, 0.33 ± 0.02, 0.56 ± 0.03 vs. -0.27 ± 0.04, all with *P* = 0.000).

**Fig. 3:**
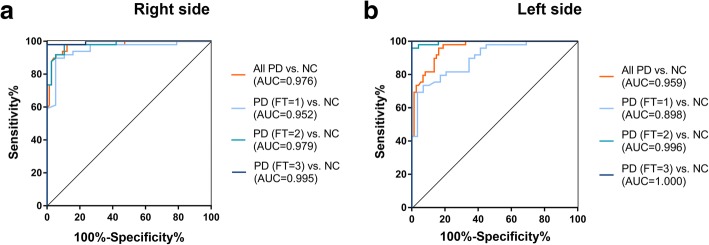
PD-Monitor FT objective score detected different severity of bradykinesia. The ROC curves illustrated strong separation between overall PD and NC, as well as between each subgroup (FT = 1, FT = 2, FT = 3) of PD and NC. **a** Right affected side, All PD vs. NC: AUC = 0.976, accuracy = 93.5%, sensitivity = 94.6%, specificity = 91.8%, cutoff = 0.018; PD (FT = 1) vs. NC: AUC = 0.952, accuracy = 89.7%, sensitivity = 94.7%, specificity = 89.8%, cutoff = 0.005; PD (FT = 2) vs. NC: AUC = 0.979, accuracy = 94.3%, sensitivity = 89.5%, specificity = 98.0%, cutoff = 0.118; PD (FT = 3) vs. NC: AUC = 0.995, accuracy = 98.5%, sensitivity = 100%, specificity = 98.0%, cutoff = 0.122; all with *P* = 0.000. **b** Left affected side: All PD vs. NC: AUC = 0.959, accuracy = 88.6%, sensitivity = 85.1%, specificity = 91.8%, cutoff = 0.072; PD (FT = 1) vs. NC: AUC = 0.898, accuracy = 81.0%, sensitivity = 65.5%, specificity = 89.8%, cutoff = 0.060; PD (FT = 2) vs. NC: AUC = 0.996, accuracy = 97.3%, sensitivity = 96.0%, specificity = 98.0%, cutoff = 0.122; PD (FT = 3) vs. NC: AUC = 1.000, accuracy = 100%, sensitivity = 100%, specificity = 100%, cutoff = 0.308; all with *P* = 0.000. PD, Parkinson’s disease; NC, normal controls; FT, finger tapping; ROC, Receiver operating characteristics; AUC, area under the ROC curve

The ROC curves illustrated strong separation between overall PD and NC, as well as between each subgroup of PD and NC (Table 2), with AUC values of 0.976, 0.952, 0.979, 0.995 (all with *P* = 0.000) for the right affected sides (Fig. 3a) and AUC values of 0.959, 0.898, 0.996, 1.000 (all with *P* = 0.000) for the left affected sides (Fig. 3b). For the right affected side, cutoff values of 0.018, 0.005, 0.118 or 0.122 could discriminate corresponding groups of PD from NC with 93.5%, 89.7%, 94.3% or 98.5% of accuracy, 94.6, 94.7, 89.5% or 100% of sensitivity and 91.8, 89.8, 98.0% or 98.0% of specificity (Fig. 3a). For the left affected side, cutoff values of 0.072, 0.060, 0.122 or 0.308 could discriminate corresponding groups of PD from NC with 88.6, 81.0, 97.3% or 100% of accuracy, 85.1, 65.5, 96.0% or 100% of sensitivity and 91.8, 89.8, 98.0% or 100% of specificity (Fig. 3b).

**Table 2:** PD-Monitor FT objective score differentiated PD from NC

	Right side					Left side				
	AUC	Sens %	Spec %	Acc %	Cutoff	AUC	Sens %	Spec %	Acc %	Cutoff
All PD vs.NC										
	0.976	94.6	91.8	93.5	0.018	0.959	85.1	91.8	88.6	0.072
Subgroups of PD vs. NC										
FT 1 vs.NC	0.952	94.7	89.8	89.7	0.005	0.898	65.5	89.8	81.0	0.060
FT 2 vs.NC	0.979	89.5	98.0	94.3	0.118	0.996	96.0	98.0	97.3	0.122
FT 3 vs.NC	0.995	100	98.0	98.5	0.122	1.000	100	100	100	0.308
H-Y 1 vs.NC	0.963	94.4	91.8	92.5	0.029	0.899	66.7	98.0	89.6	0.141

*AUC* the area under the receiver operating characteristic curve, *Sens* Sensitivity, *Spec* Specificity, *Acc* accuracy, *PD* Parkinson’s disease, *NC* normal controls, *FT* finger tapping, *H-Y* modified Hoehn and Yahr stage

### PD-monitor FT objective score could be used for early diagnosis of PD

**Fig. 4:**
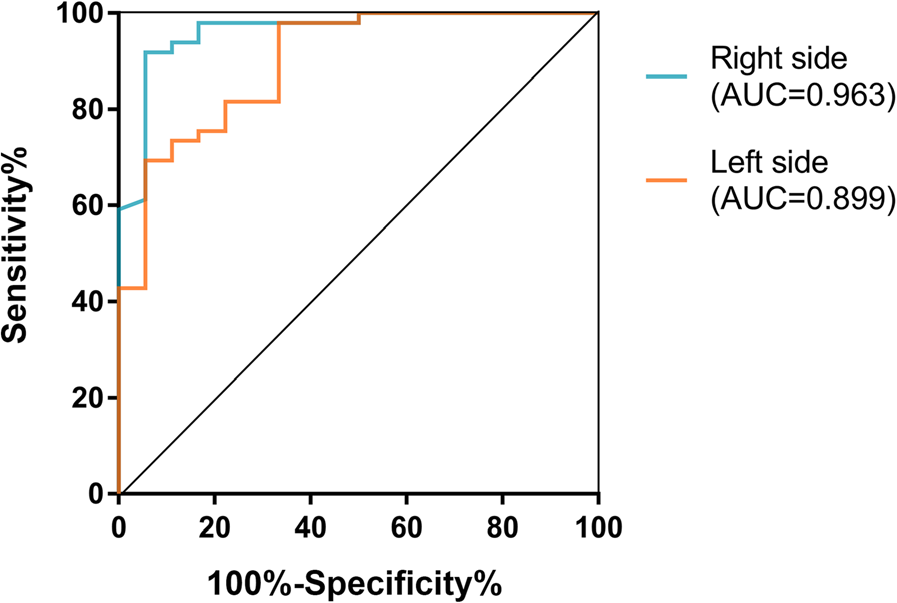
PD-Monitor FT objective score could potentially discriminate early stage PD from NC. The ROC curves illustrated a strong separation between the early PD patients (H-Y 1) and NC. Right affected side: AUC = 0.963, *P* = 0.000, accuracy = 92.5%, sensitivity = 94.4%, specificity = 91.8%, cutoff value = 0.029; Left affected side: AUC = 0.899, *P* = 0.000, accuracy = 89.6%, sensitivity = 66.7%, specificity = 98.0%, cutoff value = 0.141. PD, Parkinson’s disease; NC, normal controls; H-Y, Hoehn and Yahr stage; ROC, Receiver operating characteristics; AUC, area under the ROC curve

In order to identify whether the PD-Monitor FT objective score could potentially discriminate early stage PD from NC, we compared FT scores of 36 early stage PD patients (whose H-Y stage was 1 and FT score above 0) with 49 age and gender matched NC (Table 1). It turned out that PD-Monitor scores were significantly higher in early PD patients than that in NC (right affected side: 0.28 ± 0.05 vs.-0.24 ± 0.03, *P* = 0.000; left affected side: 0.21 ± 0.06 vs. -0.27 ± 0.04, *P* = 0.000). The ROC curves illustrated a strong separation between the early PD patients and NC (Table 2), with an AUC of 0.963 (*P* = 0.000) equivalent to accuracy/sensitivity/specificity of 92.5%/94.4%/91.8% at the cutoff value of 0.029 for the right affected side, and an AUC of 0.899 (*P* = 0.000) equivalent to accuracy/sensitivity/specificity of 89.6%/66.7%/98.0% at the cutoff value of 0.141 for the left affected side (Fig. 4).

### PD-monitor FT objective scores could differentiate ET without bradykinesia from PD

Forty-one ET patients were recruited in the present study. Ten ET patients were rated as 1 with MDS-UPDRS FT in either right or left or both hands, where 7 were scored 1 in the right hands, and 6 were scored 1 in the left hands. Using the cutoff values for discriminating overall PD from NC, that is 0.018 for the right side and 0.072 for the left side, 87.1% (27/31) ET without bradykinesia were differentiated from PD in the right side hands with 94.6% of specificity and 92.4% of accuracy; while 90.3% (28/31) ET without bradykinesia were differentiated from PD in the left side hands with 85.3% of specificity and 86.8% of accuracy. However, for those ET with MDS-UPDRS FT scored 1, 5 out of 7 (71.4%) or 3 out of 6 (50.0%) were recognized as objective bradykinesia in the right or left hand sides, indicating that they could not be differentiated from PD bradykinesia.

### The relationship between PD-monitor FT objective score and disease severity

Modified H-Y staging was used to classify PD patients into three subgroups. For the right affected side, 18 PD patients were at H-Y stage 1, but assigned as H-Y 1–1.5, 28 were at H-Y 2–2.5, and 5 were at H-Y 3–4. For the left affected side, 18 were at H-Y stage 1, 27 were at H-Y 2–2.5, and 9 were at H-Y 3–4.

Results showed that the PD-Monitor score gradually increased as modified H-Y stage increased in both affected hand sides (right affected side: 0.28 ± 0.05 in H-Y 1–1.5 vs. 0.34 ± 0.04 in H-Y = 2–2.5 vs. 0.41 ± 0.07 in H-Y = 3–4, *P* = 0.583; left affected side: 0.21 ± 0.06 in H-Y 1–1.5 vs. 0.35 ± 0.04 in H-Y = 2–2.5 vs. 0.52 ± 0.05 in H-Y = 3–4, *P* = 0.007). However, the significance was only achieved between H-Y 1–1.5 and H-Y 3–4 in the left affected side (*P* = 0.005). Correlation analysis also showed that the PD-Monitor score was related with modified H-Y stage in the left side (*r* = 0.452, *P* = 0.001). Partial correlation analysis further revealed this positive correlation still existed even after controlling age, gender, disease duration and LEDD (*r* = 0.388, *P* = 0.008).

## Discussion

This is the first study to validate evolutionary algorithms in a Chinese population. In our study, we included different grades of bradykinesia from 0 to 3 rated by MDS-UPDRS FT item, and found a strong correlation between PD-Monitor FT objective scores and MDS-UPDRS FT subjective scores. Furthermore, we demonstrated that using different cutoff values, PD-Monitor could discriminate different severity of bradykinesia from normality with high accuracy, sensitivity and specificity, which not only confirmed the results of the first study on PD-Monitor which only included PD patients with slight bradykinesia [9, 15], but also provided an evidence for a wider application of the device assessing slight to moderate bradykinesia. It is also worthy to note that in our present study, all participants were right-hand dominant and the PD-Monitor FT data from the affected limbs of patients were compared with that of the same sides from NC, making the design more rigorous than the first study [9, 15]. Indeed, in our study, different cutoff values were found between two hand sides for identifying the same degree of subjective bradykinesia. Overall, the cutoff values were larger in the left hand side than that in the right hand side based on their similar specificity, whereas the sensitivity compromised in the left sides.

Our present study also demonstrated that PD-Monitor could differentiate PD from ET without bradykinesia with high accuracy, sensitivity and specificity. However, this discrimination seemed to rely on the existence of bradykinesia itself, rather than the different nature of bradykinesia, since the present EAs could not differentiate the FT bradykinesia pattern of ET from that of PD. Slower movements could be observed in certain ET patients [5, 17–19], whereas reduction in amplitudes or freezing has not been reported in ET, which indicated that bradykinesia features of ET was probably different from that of PD. Because the EA used in this study was trained by using FT kinetics recordings of PD and NC [9], not PD and ET with bradykinesia, it would limit its capability of differentiating various bradykinesia patterns of FT.

Furthermore, our study suggested that PD-Monitor had potential to be used for diagnosis of early stage PD with high accuracy, sensitivity and specificity in the right dominant affected side. This has not been reported before, but it was consistent with the results that it could accurately detect slight bradykinesia, which was found both in our present study and the previous study [9, 15]. A positive correlation between PD-Monitor score and modified H-Y stage was also found in our cohort. The correlation of FT objective assessment and disease severity in PD was also reported in previous studies [20–22], in which they used UPDRS motor scores, a more reliable marker to assess the disease severity. However, in our study, this correlation was only found in the left affected side. The precise reason is unknown, but small samples of subgroups might be one factor affecting the statistical power.

Taking together, our study presented the value of PD-Monitor in clinical practice. Other research groups have also explored computer-based systems and wearable sensors to quantitatively assess parkinsonism related symptoms including bradykinesia [23–28] and dyskinesia [29, 30]. Different tasks were used to assess bradykinesia such as FT [27], hand movements [28], toe tapping and leg agility [29]. In general, most of them only focus on gross movement features, such as mean amplitude and velocity, and construct the classifier through linear combinations of those features [26]. In contrast, EAs produce dynamical classifiers which are particularly useful for the problems where classification involves complex, dynamical, and poorly understood modeling processes [9]. In this regard, EAs appear to be a preferred method used in neurological diagnosis.

Limitations of this study are as follows. First of all, the present study was a cross-section study, and the raters were not blind to the diagnosis of subjects. Secondly, the PD patients assessed in this study were allowed to take their usual medication. Although it did not affect the evaluation of relationship between PD-Monitor FT score and MDS-UPDRS FT score, it might impact on the relationship between PD-Monitor FT score and disease severity. Finally, due to the small sample size of ET with bradykinesia, it is too early to conclude that PD-Monitor could not differentiate ET with bradykinesia from PD. In the future, more research is needed to test the differential capability of PD-Monitor for various bradykinesia patterns of FT. The spectrum of diseases could include ET with bradykinesia, atypical parkinsonian syndromes, or other neurological diseases with slowness of finger movements such as stroke or motor neuron diseases.

## Conclusions

Our study demonstrates that a simple to use device employing classifiers derived from EAs could not only be used to accurately measure different severity of bradykinesia in PD, but also has the potential to differentiate early stage PD from normality.

## Abbreviations

AUC: Area under the ROC curve
EA: Evolutionary algorithm
ET: Essential tremor
FT: Finger-taping
H-Y: Modified Hoehn and Yahr stage
LEDD: Levodopa equivalent daily dose
MDS-UPDRS: Movement Disorder Society-sponsored revision of the UPDRS
NC: Normal controls
PD: Parkinson’s disease
ROC: Receiver operating characteristics
UK: United Kingdom
UPDRS: Unified Parkinson’s disease rating scale
